# Selected Approaches to Disrupting Protein–Protein Interactions within the MAPK/RAS Pathway

**DOI:** 10.3390/ijms24087373

**Published:** 2023-04-17

**Authors:** Stephen J. Harwood, Christopher R. Smith, J. David Lawson, John M. Ketcham

**Affiliations:** Mirati Therapeutics, 3545 Cray Court, San Diego, CA 92121, USA

**Keywords:** MAPK, KRAS, RAS, protein–protein interactions, X-ray crystallography

## Abstract

Within the MAPK/RAS pathway, there exists a plethora of protein–protein interactions (PPIs). For many years, scientists have focused efforts on drugging KRAS and its effectors in hopes to provide much needed therapies for patients with *KRAS*-mutant driven cancers. In this review, we focus on recent strategies to inhibit RAS-signaling via disrupting PPIs associated with SOS1, RAF, PDEδ, Grb2, and RAS.

## 1. Introduction

Critical cellular processes from signal transduction to genetic expression involve key interactions between proteins. Known as the interactome [1,2,3,4], this vast and crucial network of protein–protein interactions (PPIs) represents an alluring mechanism of action for targeting numerous human illnesses through the disruption of these natural associations of proteins. Disrupting these interactions potently and selectively with drug molecules suitable for oral administration can be a difficult feat. On average, the interaction surface area between two proteins is 1500 Å^2^ and can be as large as 6000 Å^2^. These surfaces are frequently devoid of well formed binding pockets, channels, and grooves in which small molecules typically bind [5,6,7,8,9,10,11], which are typically on the order of 10^2^–10^3^ Å^2^ [6,12]. Successful inhibitors usually target critical regions within the interaction surface area, known as hot spots, where proteins make essential and strong associations through the interactions of their respective amino acids [5,13,14]. PPI inhibitors routinely fall outside of Lipinski physiochemical property space [15] by having higher molecular weights and LogP values than the average approved small molecule drug [16,17]. Protein–protein interactions and dynamics [7,8,9,18,19], PPI inhibitors and activators [13,17,20,21,22,23,24,25], strategies for discovering small molecule effectors of PPIs [10,26,27,28,29], and the use of PPI inhibitors for oncology [30,31] have all been extensively reviewed previously. This review will focus on the diverse approaches used to design and develop PPI disruptors within the MAPK/RAS pathway.

**Figure 1:**
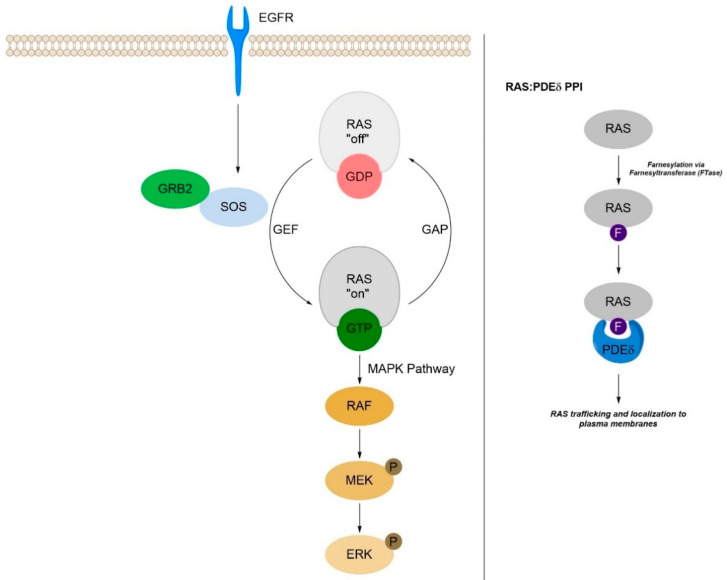
Portions of the RAS/MAPK Pathway that depict the roles of GRB2, SOS1, RAS, RAF, and PDEδ (Figure modified from Malek et al. [34]).

Activating mutations of *KRAS* are among the most common driver mutations in human cancers, leading to aberrant signaling and hyperactivation within the MAPK pathway. The majority of *KRAS* mutations are single codon mutations (G12, G13, Q61, etc.), and show a high occurrence in NSCLC (non-small cell lung cancer), CRC (colorectal cancer), and pancreatic cancers [32,33,34,35]. Due to the high prevalence of *KRAS* mutations in human cancers, these mutations have become a major focus for countless researchers for over forty years. Until the recent clinical approval of the *KRAS*^G12C^ -targeted-compounds adagrasib (MRTX849) [36,37,38] and sotorasib (AMG510) [39,40], *KRAS* was thought to be an undruggable protein. Adagrasib and sotorasib are irreversible binders of KRAS^G12C^ that form a covalent bond with the G12C mutant residue. Extensive work is ongoing to discover additional KRAS drugs that bind to other *KRAS* mutant proteins. Recently, Mirati Therapeutics announced the IND clearance for MRTX1133, an orally dosed, selective KRAS^G12D^ inhibitor [41,42]. In parallel to the considerable efforts to discover small molecules that target the mutant forms of the KRAS proteins, alternative approaches have also focused on the removal of KRAS and other proteins within the MAPK pathway (such as SOS1 PROTACS [43]) via protein degradation [44,45,46,47,48]. For this review, we focus the discussion on four protein–protein interactions that can directly impact the RAS pathway, SOS1:RAS, RAS:RAF, PDEδ:RAS, and SOS1:Grb2, as well as KRAS-targeted compounds that impact its interactions with various binding partners (Figure 1). The protein interactions described herein have been the focus of several recent drug discovery efforts within the RAS/MAPK pathway, making them attractive subject matter for this review.

## 2. Inhibitors of the SOS1:RAS PPI

**Figure 2:**
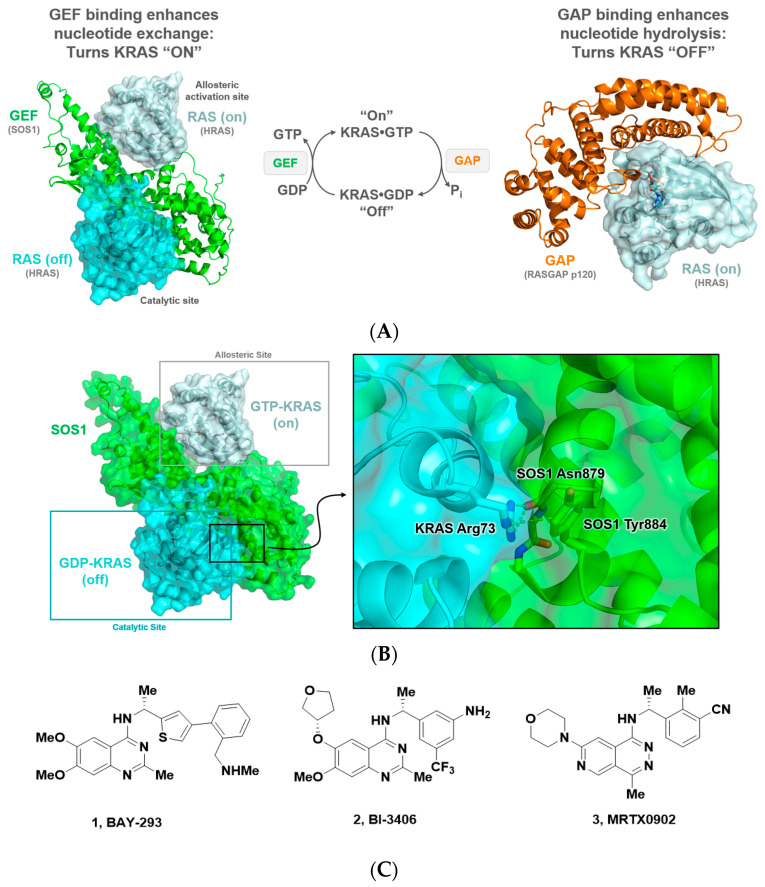
(**A**) Depiction of GEF and GAP activity to convert KRAS between its GDP- and GTP-loaded states. (**B**) SOS1 in green, in cyan GDP-KRAS binding to the catalytic site on SOS1, and in grey GTP-KRAS binding to SOS1 in an allosteric binding site. Key protein–protein interactions between KRAS Arg73 and SOS1 Asn879 and Try884 are highlighted. (**C**) Selected compound structures of SOS1 binders that disrupt the SOS1:RAS PPI including: BAY-293 (**1**), BI-3406 (**2**), and MRTX0902 (**3**).

The Son of Sevenless (SOS) proteins are guanine nucleotide exchange factors (GEFs) which play a crucial role in cellular signaling by catalyzing the nucleotide exchange of GTPase-activating proteins such as RAS (Figure 2A) [49,50,51,52]. As a result, this protein–protein interaction has garnered significant interest as a potential target within the MAPK/RAS pathway for small molecule therapeutics. Key interactions between SOS1 and KRAS can be found within the catalytic site of SOS1, where a GDP-loaded KRAS (cyan, Figure 2B) binds via the KRAS:SOS1 protein–protein interaction between Arg73 of KRAS and Asn879/Tyr884 of SOS1 (Figure 2B). An additional KRAS molecule, this one in its GTP-loaded on state, binds to an allosteric pocket on the opposite side of SOS1 and is believed to help accelerate the turnover and activation of RAS in the catalytic pocket. This results in a positive feedback loop for the RAS pathway. The well-defined PPI between SOS1 and KRAS has made it a highly sought-after target to lock KRAS into its off state, in an attempt to shut down or slow the aberrant signaling caused by mutant forms of KRAS. To this end, numerous groups have focused on the design of compounds that bind to the SOS1 protein and disrupt the PPI between KRAS:SOS1. Selected examples of such binders are shown in Figure 2C (BAY-293, BI-3406, and MRTX0902; **1**–**3**).

### 2.1. Discovery of BAY-293

**Figure 3:**
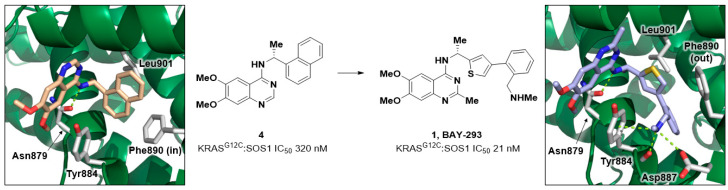
Compound structure of HTS hit **4,** leading to the discovery of BAY-293 (**1**). X-ray co-crystal structure of **4** bound to SOS1 (PDB 5OVE). A key hydrogen bond between the aniline N-H of **4** and the side chain of Asn879 was observed and the naphthyl group **4** was bound in a hydrophobic pocket formed by Leu901 and Phe890. X-ray co-crystal structure of **1** bound to SOS1 (PDB 5OVI). Phe890 side chain rotates from the back of the pocket towards the front of the pocket. The basic amine makes an ionic interaction with Asp887, a hydrogen bond with the backbone carbonyl of Tyr884, and a cation-π interaction with the sidechain of Tyr884.

The discovery of BAY-293 (**1**) started with a high throughput screen of over 3 million compounds using an enzymatic HTRF SOS1 nucleotide exchange assay to identify quinazoline hit **4** with an IC_50_ of 320 nM (Figure 3). Through a series of biophysical methods, it was determined that **4** bound to SOS1 and not to KRAS^WT^ or KRAS^G12C^. [53] To understand the binding mode, a co-crystal structure of **4** bound to SOS1 was obtained. The inhibitor binds in a well-defined pocket within the catalytic region of SOS1—a region important for KRAS binding. A key hydrogen bond between the anilinic N-H of **4** and the side chain of Asn879 was observed, and the naphthyl group of **4** filled in a hydrophobic pocket formed by Leu901 and Phe890. Subsequent optimization supported by X-ray crystallography resulted in the discovery of BAY-293 (**1**) with an IC_50_ of 21 nM in an SOS1:KRAS interaction assay. The X-ray co-crystal structure of BAY-293 (**1**) revealed that the binding mode was closely related to that of the initial HTS hit **4**; however, the Phe890 side chain was found to rotate from a “Phe890-in” conformation towards the front of the pocket in a “Phe890-out” pose. The movement of the Phe890 helped to accommodate the phenyl ring at the 4-position of the thiophene and resulted in the aminomethyl group forming two hydrogen bonds with Tyr884 and Asp887. In K-562 cells, **4** was shown to modulate pERK levels without impacting the parent ERK protein levels. For these quinazoline-based SOS1 binders, the 6-methoxy substituent extends from the SOS1 binding pocket and effectively blocks the KRAS:SOS1 PPI.

### 2.2. Discovery of BI-3406

**Figure 4:**
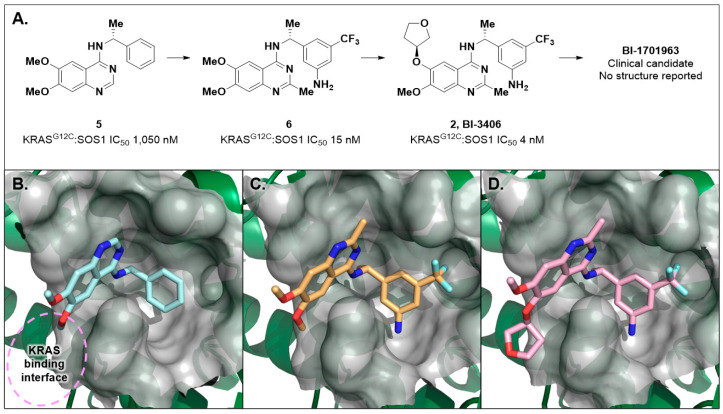
(**A**) Optimization of HTS hit **5** through **6** and BI-3406 (**2**) to identify the clinical candidate BI-1701963. (**B**) X-ray co-crystal structure of **5** bound to SOS1 (PDB: 6SFR) displays the same binding mode as **4** (Figure 3). (**C**) X-ray co-crystal structure of **6** bound to SOS1 (PDB: 7AVU). Note the Phe890 side chain prefers the Phe890-in conformation. The primary anilino group makes a productive hydrogen bond with the Met878 backbone carbonyl. (**D**) X-ray co-crystal of BI-3406 (**2**) bound to SOS1 (PDB: 6SCM). The tetrahydrofuran moiety is projected into space where KRAS binds to SOS1, thus disrupting the SOS:KRAS PPI.

During their pursuit of a clinical SOS1 binder, researchers at Boehringer Ingelheim screened 1.7 million compounds using a KRAS^G12D^-biotin GST-SOS1 protein–protein interaction assay via AlphaScreen and fluorescence resonance energy transfer (TR-FRET) readouts [54]. Serendipitously, this screening identified a similar quinazoline scaffold, as was found through the Bayer efforts, identifying hit **5** with an IC_50_ of 1050 nM (Figure 4A). Notably, **5** and **4** share the same quinazoline scaffold and differ only by naphthyl in **4** being swapped out for a phenyl substituent in **5**. X-ray crystallography revealed **5** bound to SOS1 with the same binding mode as observed with **4** (Figure 4B). Subsequent optimization of **5**, supported by X-ray crystallography, identified aniline **6** SOS1:KRAS IC_50_ = 15 nM (Figure 4A,C). Further in vitro and in vivo PK profiling led to the identification of the tool compound BI-3406 (**2**, Figure 4A,D). BI-3406 displayed modulation of pERK and antiproliferative properties in NCI-H23 cells with either a G12C, G12D, G13D, G12V, or G12R mutation of *KRAS*. Additionally, BI-3406 (**2**) demonstrated 87% in vivo tumor growth inhibition (TGI) when dosed as a single agent at 50 mg/kg bid over 35 days in an MIA PaCa-2 (human tumor cell line) mouse xenograft tumor model [55]. Although the structure has not been reported, BI-1701963 was selected as a clinical-stage inhibitor of the SOS1:KRAS PPI, and has displayed tumor regressions in several tumor models in mice when dosed in combination with a KRAS^G12C^ inhibitor. BI-1701963 entered clinical studies in October of 2019 (NCT04111458), and clinical studies have focused on dosing alone and in combination with other RAS pathway modifiers in patients with solid tumors containing *KRAS* mutations. To date, while no clinical updates have been reported for BI-1701963, the study (NCT04111458) is no longer recruiting and appears to have been stopped during the early parts of Phase 1.

### 2.3. Discovery of MRTX0902

**Figure 5:**
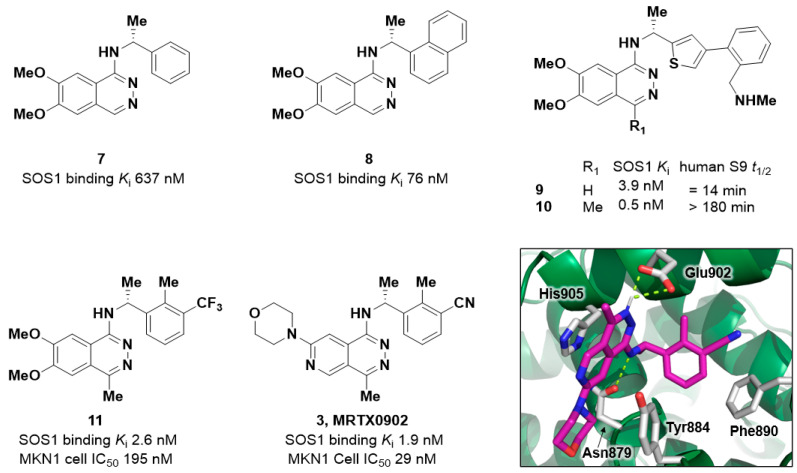
Exemplar compounds highlighting the potency and physiochemical property optimization of the phthalazine scaffold, resulting in the discovery of MRTX0902 (**3**). X-ray co-crystal structure of MRTX0902 bound to SOS1 (PDB 7UKR).

Molecular modeling of the SOS1 X-ray co-crystal structures of **4** and **5** suggested that transposition of the N1-quinazoline nitrogen to form a phthalazine scaffold would retain SOS1 binding activity (Figure 5). Remarkably, in an HTRF displacement binding assay the corresponding phthalazine analogs **7** and **8** resulted in nanomolar binding activity (SOS1 *K*_i_ = 76 nM and 637 nM, respectively) [56]. Analogous to BAY-293 (**1**), [53] the phthalazine analog **9** (SOS1 *K*_i_ = 3.9 nM) was prepared, and although it is a potent binder of the SOS1 protein, **9** was highly susceptible to oxidative metabolism by aldehyde oxidase (AO). This compound presented with a short half-life in human liver S9 liver fractions (*t*_1/2_ = 14 min). A C4-methyl substituent was installed to give compound **10**, which successfully blocked the AO-mediated metabolism in human liver S9 liver fractions (*t*_1/2_ > 180 min) and resulted in an increase in binding potency (SOS1 *K*_i_ = 0.5 nM). Further optimization identified compound **11** that demonstrated inhibition of ERK1/2 phosphorylation, detected via in-cell Western assay, with an IC_50_ of 195 nM in the MKN1 cell line. Installation of a C7-morpholine and a cyano group on the benzylamine led to the optimization of physiochemical and ADME properties, resulting in the discovery of the clinical-stage SOS1 binder MRTX0902 (**3**, SOS1 *K*_i_ = 0.5 nM, MKN1 cellular IC_50_ = 29 nM).

The co-crystal structure of MRTX0902 bound to SOS1 (PDB 7UKR) is shown in Figure 5. The key interactions include a π-stacking interaction between His905 and the phthalazine core, a crucial hydrogen bond between Asn879 and the N-H of the C1-benzyl amine, the α-methyl on the benzylic amine fills a small cavity in the pocket and positions the phenyl group for an edge-to-face interaction with Phe890, and the C7-morpholine protrudes out of the binding pocket and into the region where the SOS1:KRAS PPI occurs, driving the disruption of the SOS1:KRAS complex.

In the MIA PaCa-2 (KRAS^G12C^) mouse xenograft tumor model, when dosed orally (50 mg/kg bid for 25 days dosing) MRTX0902 demonstrated 53% TGI. [56] In the same model, a combination of MRTX0902 (50 mg/kg, bid) with a sub-optimal oral dose of the KRAS^G12C^ inhibitor MRTX849 (10 mg/kg, qd) demonstrated near complete tumor regression (−92%, two tumor free animals). A phase 1/2 clinical study of MRTX0902 in solid tumors with mutations in the MAPK/KRAS pathway was recently initiated (NCT05578092) and includes a combination arm with adagrasib (MRTX849).

BAY-293, BI-3406, and MRTX0902 are examples of small molecule PPI inhibitors within the Lipinski’s “Rule-of-five” physicochemical property space. Presumably, the presence of a well formed binding pocket on the SOS1 surface and the co-location of vital hot-spot interactions between SOS1 and KRAS in the vicinity of the binding pocket aided in the discovery of BAY-293, BI-3406, and MRTX0902.

## 3. Disrupting the SOS1:Grb2 PPI

Although the SOS1:Grb2 PPI is not a direct PPI with RAS, we include it in this review because SOS1 plays a critical role in the turnover of KRAS from its GDP-loaded “off” state to its GTP-loaded “on” state. Perturbing SOS1 activity can in turn disrupt KRAS signaling and, therefore, an SOS:Grb2 PPI inhibitor may play an important role in a drug combination strategy for the treatment of *KRAS* mutant driven cancers.

**Figure 6:**
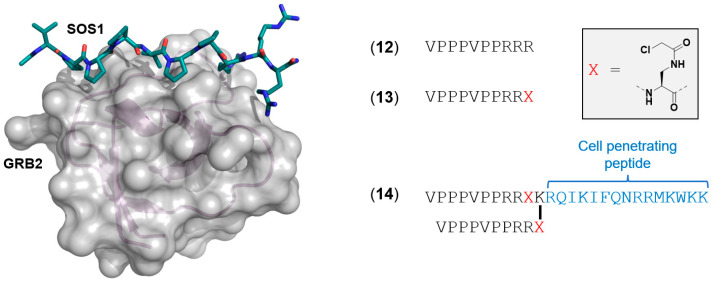
Solution NMR Structure of GRB2 with an SOS1 ten-residue peptide (PDB 1GBQ) [61]. Sequences used in developing the covalent binding, cell penetrant peptide **14** [62]. Peptide sequences shown without capping groups for clarity.

The growth factor receptor bound protein-2 (Grb2) is an adaptor protein that recruits SOS1 from the cytosol to the plasma membrane where SOS1 binds to RAS. The Grb2–SOS1 interaction is mediated between the N- and C-terminal SH3 domains (nSH3/cSH3) of Grb2 and the proline-rich (PR) domain of SOS1. [57,58,59,60] In the late 1990s, scientists at Bristol-Myers Squibb (BMS) disclosed a solution NMR structure of a ten-residue peptide (ac-VPPPVPPRRR-NH_2_, **12**) derived from SOS1 (residues 1135 to 1144) complexed with the nSH3 domain of Grb2 (Figure 6). [61] This highly truncated peptide of SOS1 (**12**) was reported to have a K*_D_* of 3.5 µM binding to N-terminal SH3 domain Grb2 (Grb2 ^N-SH3^, N-terminal Src homology 3 domain) [61]. Xia et al. used peptide **12** as a foundation for designing a potential covalent modifier of the Grb2:SOS1 PPI [62]. The close proximity of the N-terminus of **12** to a surface exposed cysteine residue (Cys32) on Grb2 allowed for replacement of the C-terminus arginine of **12** with an electrophilic chloroacetyl functional group to give **13** (Figure 6, diaminopropionic acid with a chloroacetyl group, Dap (Cl-Ac)). Covalent binding of **13** to Grb2 ^N-SH3^ was first demonstrated via SDS-PAGE and MALDI-TOF MS analysis, revealing the formation of the Grb2^N-SH3^-**13** covalent complex. The authors also monitored the kinetics for the formation of the covalent adduct between full length Grb2 with **13** and found the complex to have a *k*_app_ = 1.87 ± 0.03 h^−1^. Further optimization of **13** included dimerization and installation of a cell-penetrating peptide (CPP) sequence to form peptide **14**. Treating Grb2-transfected COS-7 cells with **14** resulted in the formation of the Grb2-**14** adduct within the cells. Additionally, when **14** was used to treat SKBR3 cells (breast cancer cell line), the peptide was able to inhibit cell migration, ERK phosphorylation, and cellular viability. The cell penetrant peptide **14** is a useful irreversible probe compound to study the SOS1:Grb2 complex and presents as an interesting strategy for indirectly modulating RAS activity.

## 4. Targeting the RAS:RAF PPI

**Figure 7:**
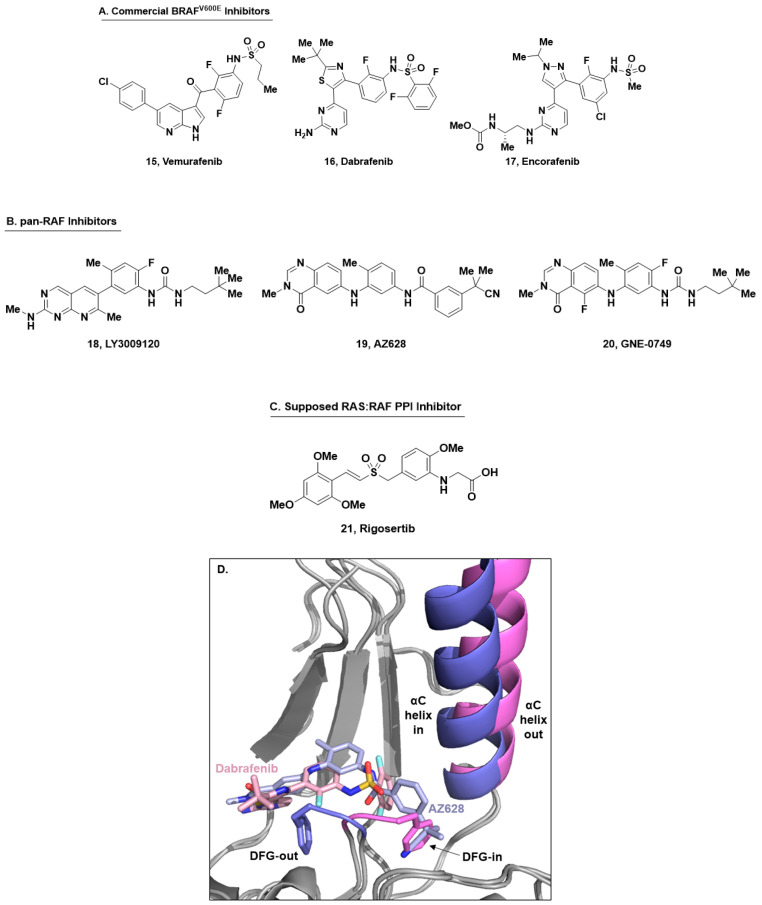
(**A**) Compound structures of commercial B-RAF^V600E^ inhibitors vemurafenib, dabrafenib, and encorafenib (**15**–**17**). (**B**) Selected pan-RAF inhibitors LYS3009120, AZ628, and GNE-0749 (**18**–**20**). (**C**) Chemical structure of rigosertib (**21**), the supposed RAS:RAF PPI inhibitor. (**D**) Comparison of X-ray crystal structures of dabrafenib (**16**, in pink) and AZ628 (**19**, in blue) bound to BRAF^V600E^ showing ligand-induced conformational changes of the DFG-motif and αC-helix. The DFG-out/αC-helix-in conformation prevents dimerization under activated RAS conditions.

RAS in its active, GTP-bound, form promotes the dimerization and phosphorylation of the RAF family of kinases (A-RAF, B-RAF, and C-RAF), which in turn leads to the phosphorylation of MEK1/2 and further downstream signaling within the RAS/MAPK pathway [63,64]. The discovery of B-RAF^V600E^ inhibitors that target the orthosteric/ATP-binding site has resulted in three approved B-RAF^V600E^ inhibitors: vemurafenib [25,65,66,67], dabrafenib [68,69,70,71], and encorafenib (Figure 7A, **15**–**17**) [72,73,74]. Over time, however, treatment with B-RAF^V600E^ inhibitors has given rise to acquired resistance mechanisms within treated patients [75,76,77]. Additionally, B-RAF^V600E^ inhibitors surprisingly activate the MAPK pathway in KRAS mutant driven cancers [78]. It has since been established that the binding mode of RAF inhibitors is crucial for controlling the hetero-/homodimerization of RAF proteins that results in activation of the pathway [79,80,81,82]. Specifically, B-RAF^V600E^ inhibitors can increase dimerization potential with uninhibited RAF monomers in the cell, thus driving pathway activation when KRAS is in its active form. Recently, it has been demonstrated that type II “pan-RAF” inhibitors, such as LY3009120 [83,84,85,86], AZ628 [87,88], and GNE-0749 [89] (Figure 7B, **18**–**20**), do not cause activation of the MAPK pathway [63]. This outcome is driven by an alternative RAF protein confirmation where the αC helix is “in” and the DFG activation loop is “out”, leading to the formation of hetero- and homodimers of RAF and inhibition of downstream signaling. In addition to BRAF^V600E^ inhibition, these pan-RAF inhibitors also inhibit wild-type B-RAF and C-RAF.

An alternative approach to inhibit the action of the RAF kinases is to disrupt the protein–protein interactions between RAF and RAS. The small molecule rigosertib (Figure 7C, **21**) was originally thought to behave as an RAS mimetic, binding to the RAS binding domain (RBD) of C-RAF and B-RAF (MST K*_D_*: 0.18 and 0.71 nM, respectively) and disrupting the RAS:RAF PPI [90]. However, the mechanism of action of rigosertib has some uncertainty [91,92,93], and more recently rigosertib was characterized as a microtubule-destabilizing agent [93]. Due to the ambiguity and lack of crystallographic evidence of binding near the RAS:RAF interface, it is fair to state that no small molecule inhibitors of the RAS:RAF PPI have been described in the literature to date. However, disrupting the RAS:RAF PPI is an intriguing approach to perturbing irregular KRAS signaling, and other researchers have focused on the use of cyclic peptides [94,95] or organometallic [96] compounds to disrupt the RAS:RAF PPI. Additionally, compounds that target the RAS:RAF PPI by binding to KRAS are described in the final section of this review.

## 5. Targeting the PDEδ:RAS PPI

**Figure 8:**
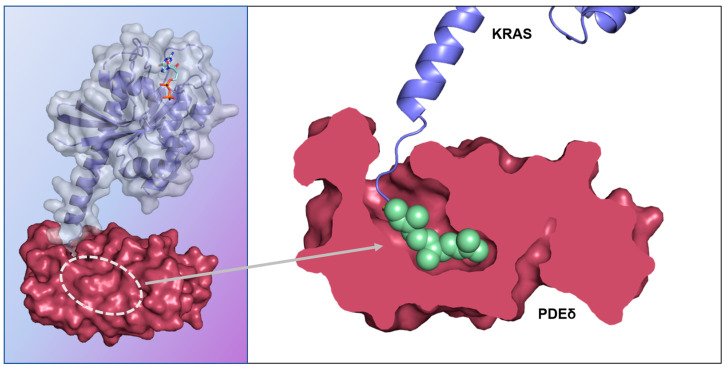
Co-complex X-ray crystal structure of KRAS (blue ribbon, grey surface) with PDEδ (red) (PDB 5TAR) [100]. The farnesylated C-terminus of KRAS (green) binds the prenyl binding pocket of PDEδ. The binding of ligands in this deep, hydrophobic pocket disrupts KRAS binding and thus its distribution within the cell.

After translation, RAS proteins are frequently modified with lipids. Broadly, there are two types of post-translational lipid modifications that occur on the RAS proteins: irreversible farnesylation and subsequent reversible palmitoylation. KRAS is not reversibly palmitoylated in its hypervariable region. Upon farnesylation, KRAS undergoes rapid mediated transport to the Golgi (and subsequently the plasma membrane). This critical spatial organization of KRAS to the plasma membrane contributes to the protein’s activity in signal transduction [97]. PDEδ binds to farnesylated KRAS and directs it to the Golgi, in doing so, PDEδ plays a pivotal role in preventing the statistical distribution of KRAS across all intracellular membranes (Figure 8). Downregulation of PDEδ has been shown to randomize the distribution of KRAS across intracellular membranes. Therefore, efforts to disrupt this spatial organization process have become an attractive target for small molecule drug discovery efforts [98,99]. In the last decade, several compounds with single digit nanomolar and even picomolar binding affinity to PDEδ have been identified.

**Figure 9:**
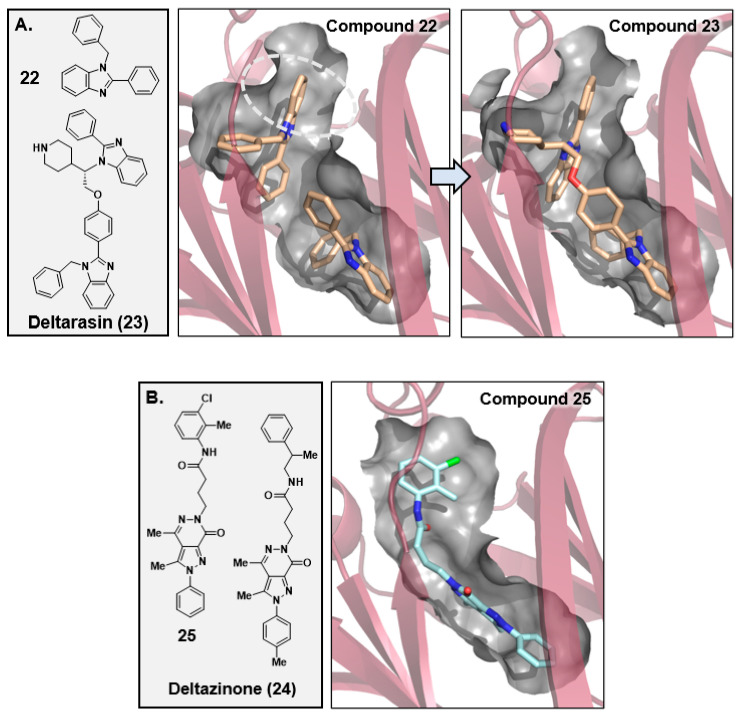

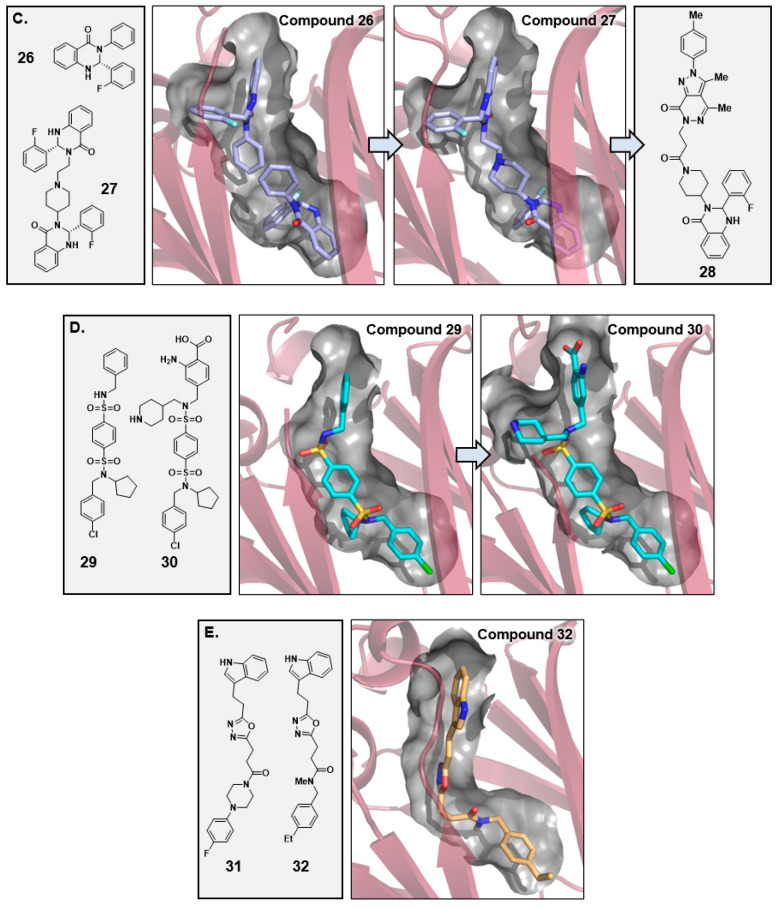
Multiple chemical series of PDEδ:RAS PPI inhibitors **22**–**32** (Figure 9**A**–**E**). In the molecular graphics, the inhibitors are shown in stick and the PDEδ protein is depicted as red ribbons. A partial surface of the PDEδ prenyl binding pocket (also see Figure 8) is shown in grey.

In 2013, Waldmann and coworkers harnessed a high-throughput Alpha Screen using farnesylated KRAS4B and His-tagged PDEδ to discover benzimidazole-based compounds that bound to the farnesyl-binding pocket of PDEδ. These compounds could effectively bind to PDEδ with nanomolar binding potencies (K*_D_*) and disrupt the protein–protein interaction between KRAS:PDEδ [101]. Hits from their initial screening efforts were further validated using a fluorescence polarization assay and isothermal titration calorimetry. An X-ray co-crystal structure was obtained and revealed that two benzimidazole fragments (**22**, Figure 9A) bound in the farnesyl-binding site of PDEδ (Figure 9A). One benzimidazole fragment bound deep in the farnesyl-binding site, while the other fragment bound closer to the opening of the hydrophobic pocket. Through rational structure-based design efforts, the two benzimidazole fragments were linked together, leading to the synthesis of deltarasin [101,102,103] (**23**, PDEδ K_D_ = 38 nM, Figure 9A), which showed a dose-dependent tumor growth inhibition of tumors in a Panc-Tu-I mouse model [101]. Through a similar screening process, Waldmann and coworkers were able to discover a new series of tumor growth inhibition of tumors in a PancTu-I mouse model [101]. Through a similar screening process, Waldmann and coworkers were able to discover a new series of pyrazolopyridazinones that could disrupt the binding between KRAS and PDEδ, and improve upon the metabolic stability and non-specific cytotoxicity of Deltarasin [104]. This led to the discovery of deltazinone (**24**, Figure 9B) and related analogs (e.g., **25**, Figure 9B). Deltazinone was evaluated in vivo and found to have low exposure through PO dosing and acceptable exposure via IP and IV administration. Sheng and coworkers later identified a unique quinazolinone fragment (**26**, Figure 9C) in a high throughput screen that, similar to the benzimidazoles, bound twice in the hydrophobic channel of PDEδ (Figure 9C) [105,106]. SBDD efforts to link these two fragments were successful, resulting in single-digit nanomolar binding of compound **27** (PDEδ K_D2_ = 2.3 nM, Figure 9C), in a fluorescence anisotropy assay. Further optimization led to replacing one of the quinazolinone moieties with the previously identified pyrazolopyridazinone [104], resulting in a compound (**28**, Figure 9C) with sub-nanomolar binding affinity (PDEδ K_D2_ = 0.6 nM).

Surprisingly, despite the potent binding affinity measured for PDEδ, these reported small molecules have required micromolar concentrations to effectively reduce cell growth. This decreased cellular potency has been attributed to a secondary PPI between PDEδ and the release factor Arl2. Under normal physiological conditions, Arl2 stabilizes PDEδ and assists in the release of KRAS from PDEδ. Despite the potent K_D_ values reported, it has been suggested that the interaction between PDEδ and Arl2 is responsible for the release of these high affinity small molecule binders in cells, thereby attenuating their cellular activity.

In 2017, Waldman and coworkers substantiated this hypothesis through a fluorescence polarization assay and reported the development of triple digit picomolar affinity binders of PDEδ that can reduce cell growth with a submicromolar IC_50_. [107] A 200,000 compound library was evaluated using an Alpha Screen where novel bis-sulfonamides (e.g., **29**, Figure 9D) were identified as potent binders of PDEδ. This fragment maintained the key H-bond interactions with Arg61, Gln78, and Tyr146 as previously found with deltazinone. Furthermore, two new interactions with the aromatic rings of Trp32 and Trp90 were proposed. The installation of a piperidine as a hydrogen bond donor enabled an interaction with the carbonyl acceptor of Cys56. An additional aniline substituent was installed to form an additional H-bonding interaction with the protein at Glu88—a total of seven H-bonds (**30**, Figure 9D).

Recently, in silico techniques have also been used to identify novel inhibitors of the PDEδ:RAS PPI. In 2022, Williams and coworkers conducted a virtual screening for novel RAC inhibitors followed by profiling these inhibitors for their ability to inhibit growth of leukemic cell lines [108]. Hit optimization efforts led to the discovery of DW0441 and DW0254 (**31** and **32**, respectively, Figure 9E), which were found to have IC_50_s in the submicromolar range when screening the viability of T-cell acute lymphoblastic leukemia cells. Serendipitously, while these molecules were originally thought to bind RAC, it was found that these compounds instead bind PDEδ. Mutagenesis of PDEδ and deletion of Arg48 and Val49 prevented the binding of DW0254 and induced resistance to the compound. Furthermore, the binding mode of DW0254 (**32**) was elucidated through X-ray crystallography efforts (Figure 9E).

**Figure 10:**
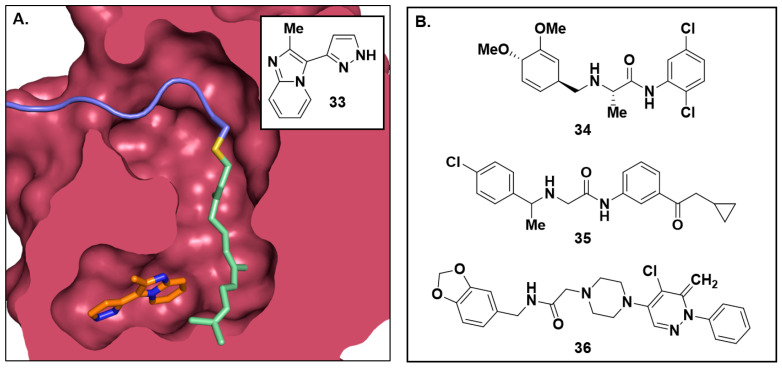
(**A**) X-ray crystal structure of imidazopyridine fragment **33** stabilizing the interaction between PDEδ and a farnesylated C-terminal peptide of KRAS (PDB 7Q9S). PDEδ is shown in red with the KRAS peptide in blue, the farnesyl group in green, and **33** in orange. (**B**) Putative KRAS/PDEδ molecular glues (**34**–**36**) identified via in silico screening techniques.

Representing a strategically different approach, in 2022, Bower and coworkers identified a small molecule fragment capable of stabilizing the PDEδ:RAS PPI, and hypothesized that this molecular glue approach could be used to disrupt KRAS spatial organization similar to the action of the PPI inhibitors [109]. Identification of this fragment hit (**33**, Figure 10A) was achieved using an SPR assay, in which an engineered high-affinity KRAS bound to PDEδ was immobilized onto an NTA chip while compounds flowed over the chip. These results were validated using ligand observed NMR (LONMR) spectroscopy, isothermal titration calorimetry, and X-ray crystallography.

Additional putative molecular glues were initially identified via in silico techniques by Vargas and coworkers. Their group harnessed virtual library screening and molecular dynamics simulations to identify aryl amide compounds (Figure 10B, **34**–**36**). When tested in vitro, these compounds were found to inhibit cellular viability in the 10–100 μM range [110,111]. However, the authors did not present evidence of direct engagement of the KRAS-PDEδ complex; thus, it is not possible to definitively conclude that these compounds behave as molecular glues. These molecules may modulate the MAPK/RAS pathway via an orthogonal mechanism, and further evidence may be needed to prove their mechanism of action.

## 6. KRAS Binders That Disrupt the PPIs between RAS and RAS Effectors

**Figure 11:**
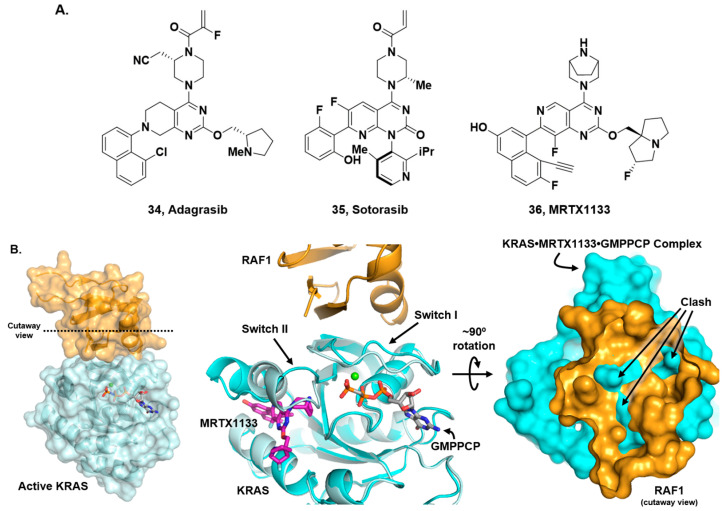
(**A**) Structures of adagrasib, sotorasib and MRTX1133 (**34**–**36**). (**B**) Left: X-ray crystal structure (PDB: 6VJJ) of the RAS binding domain of RAF1 (orange) complexed with GMPPNP-KRAS (light blue) where GMPPNP is an analog of GTP. Middle: KRAS^G12D^-MRTX1133-GMPPCP (PDB: 7T47, cyan ribbon) superposed with the KRAS-GMPPNP-RAF1 complex (light blue and orange ribbons) where GMPPCP is also an analog of GTP. Right: KRAS-RAF1 complex rotated 90° and cutting away the RAF1 surface reveals clashes with the KRAS^G12D^-MRTX1133-GMPPCP surface in cyan.

The last section of this review will address what are, perhaps, the best-known inhibitors in the MAPK/RAS pathway, KRAS “inhibitors”. It should be noted that these are not inhibitors of KRAS’s enzymatic activity. Indeed, inhibiting KRAS’s enzymatic function of hydrolyzing GTP to GDP would prevent transitioning from its ‘on’ to ‘off’ state and would thus be counter-productive to interrupting the increased signaling seen in this pathway in a cancer setting. Rather, these inhibitors disrupt the PPI between KRAS and its downstream effectors, with RAF being the most studied. Neither the approved KRAS^G12C^-targeted drugs adagrasib [36,37,38] (**34**) and sotorasib [39,40] (**35**) nor the several additional compounds working their way through the clinic behave like traditional PPI inhibitors that bind on the surface of one protein and compete with the binding of a partner protein (Figure 11A). Rather, these compounds bind in a cryptic pocket under the Switch-II loop (the Switch-II pocket), [112], thereby altering the conformation of Switch-II and local surface morphology such that KRAS is not competent to bind RAF. This change in protein structure abrogates downstream signaling, and recent work from Hallin et al. reveals that this effect is independent of the nucleotide state [42]. In contrast to adagrasib, sotorasib, and related compounds that cannot covalently bind and modify KRAS^G12C^ in the GTP-bound ‘on’ state, the KRAS^G12D^-targeted binder, MRTX1133 (**36**), can bind KRAS^G12D^ in both the GTP-bound and GDP-bound states. X-ray crystal structures show that even when in the GTP-bound state, the Switch-II loop is perturbed and is not competent to bind RAF (Figure 11B). There are currently numerous reviews [35,113,114,115] on the topic of KRAS Switch-II pocket binders; thus, we will forego a compound-by-compound dissection of this subject matter.

**Figure 12:**
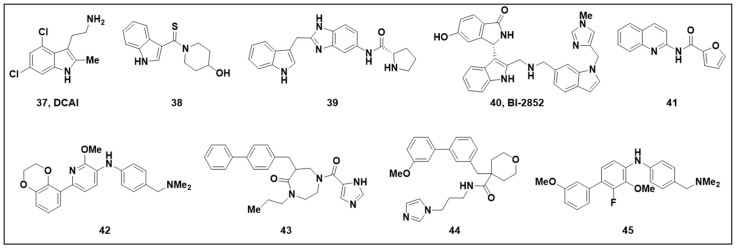
Structures of compounds (**37**–**45**).

**Figure 13:**
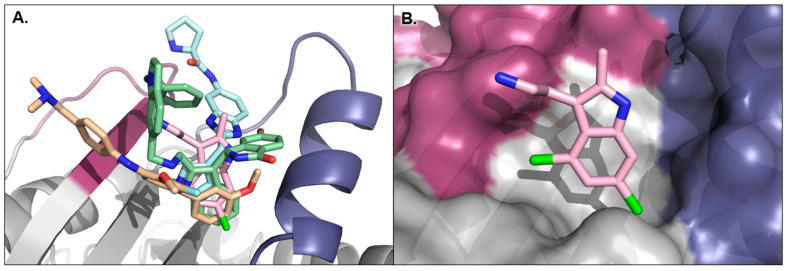

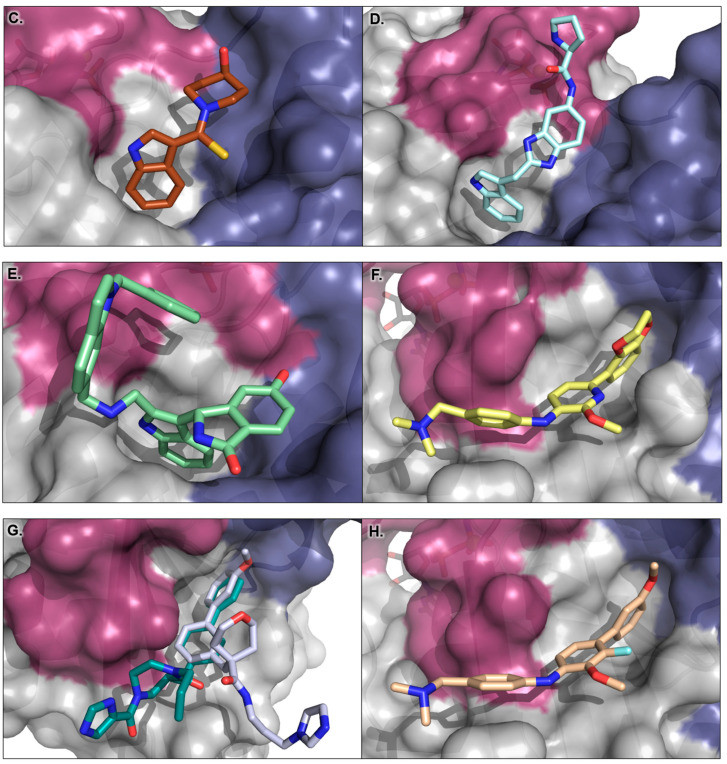
X-ray crystal structures of KRAS bound to various Switch-I/Switch-II surface pocket binders. RAS is depicted as grey with the Switch-I and Switch-II loops colored magenta and indigo, respectively. (**A**) Compounds (**37**) in pink, (**39**) in light blue, (**40**) in green, and (**45**) in orange all bind in the same pocket between the RAS Switch-I and Switch-II loops (PDBs: 4DST, 4EPY, 6GJ7, and 6GQY). For clarity, only the protein co-crystalized with (**39**) is shown. (**B**) Compound (**37**) bound to KRAS^WT^•GMPPCP (PDB 4DST). (**C**) Compound (**38**) bound to KRAS^WT^•GDP (PDB 4EPW). (**D**) Compound (**39**) bound to KRAS^WT^•GDP (PDB 4EPY). (**E**) **BI-2852** bound to KRAS^G12D^•GMPPCP (PDB 6GJ7). (**F**) Compound (**42**) bound to KRAS^Q61H^•GMPPNP (PDB 6FA4). (**G**) Overlay of compounds (**43**) in teal and (**44**) in light blue bound to KRAS^Q61H^•GMPPNP (PDBs 6GOM and 6GQT). For clarity, only the protein from the structure of (**43**) is shown. (**H**) Structure of compound (**45**) bound to KRAS^Q61H^•GMPPNP (PDB 6GQY).

While most KRAS-targeted inhibitors of MAPK/RAS pathway signaling bind in the aforementioned Switch-II pocket, there is an additional class of compounds that bind to the exterior of the protein and function like traditional PPI inhibitors, blocking KRAS effector proteins from binding. These compounds (Figure 12) map to a pocket that sits between the Switch-I and Switch-II loops on RAS (Figure 13A). One of the earliest reports mentioning this class came from scientists at Genentech, who reported that the indole fragment DCAI (**37**, Figure 12) is capable of binding KRAS, blocking SOS1 binding and concomitant nucleotide exchange [116]. The ligand was discovered in an NMR-based saturation transfer difference screen. Binding to KRAS between Switch-I and Switch-II was confirmed with protein-observed NMR in ^15^N-labeled KRAS and subsequent X-ray crystallography (Figure 13B). The compound demonstrated an IC_50_ of 342 µM in an SOS1 nucleotide-exchange assay. Around the same time, the Fesik laboratory also used NMR screening to identify several fragment-sized compounds (e.g., **38**, Figure 12) that bound multiple KRAS mutants including G12D, G12V, and wild-type [117]. X-ray crystal structures localized the compound binding to the surface depression between Switch-I and Switch-II similar to **37** (Figure 13C). Further elaborated fragments (e.g., **39**, Figure 12) demonstrated up to 78% inhibition within an SOS1 nucleotide-exchange assay, and binding was further confirmed by protein-observed NMR with ^15^N-labeled KRAS and X-ray crystallography (Figure 13D). A collaboration between the Fesik group and Boehringer Ingelheim later led to hits from additional screening combined with further SBDD optimization of the indole fragment **38**, resulting in BI-2852 (**40**, Figure 12 and Figure 13E) [118]. BI-2852 binding to KRAS^G12D^ was measured at 740 nM in the on state and 2000 nM in the off state. Additionally, BI-2852 demonstrated disruption of KRAS binding to the SOS1 nucleotide-exchange site, the SOS1 allosteric activation site, C-RAF, and PI3Kα ranging from 100 to 770 nM across KRAS^G12D^, KRAS^G12C^, and KRAS^WT^. The authors found that BI-2852 was able to inhibit pERK, pAKT, and proliferation within cellular assays in a dose-dependent manner.

An additional set of compounds that bind in the Switch-I/Switch-II surface pocket were discovered by Rabbitts et al., who performed a fragment library SPR screen against HRAS^G12V^ in both the “on” and “off” states. Screening hits were further screened for functionality via a follow-up SPR screening in the same system, to assess competition with an anti-RAS antibody fragment [119]. The antibody fragment was used because naturally occurring RAS effectors have weak binding affinities and off-rates are too fast for use in a competition experiment. The resulting weak (>370 µM) quinoline hit (**41**, Figure 12) was progressed via structure-based design using KRAS^Q61H^ and KRAS^G12D^ co-crystal structures (both in the “on” state). This resulted in the design of **42** (Figure 12 and Figure 13F), which displayed a K_D_ of 51 nM for the “on” state of KRAS^G12V^ via a waterLOGSY NMR experiment. Additionally, compound **42** showed dose-dependent effector competition in BRET-based cellular PPI assays across several RAS/effector pairs; resulting in dose-dependent inhibition of AKT and ERK phosphorylation in DLD-1 and H358 cell lines, as well as inhibition of cell viability in DLD1 and HT1080 cell lines (8 and 10 µM IC_50_s, respectively). Molecular modeling helped to explain the cellular data, by revealing that the binding of **42** to KRAS overlaps with the binding sites of effectors RAF, PI3K, and RALGDS.

In a follow-up study by the same group [120], an SPR screen with KRAS^G12V^ was used to discover two hits **43** and **44** (Figure 12 and Figure 13G) that were further confirmed through waterLOGSY NMR experiments. However, neither hit was competitive with the anti-RAS antibody fragment that was used in their previous studies [119]. By computationally aligning the 3D structures of the hits with the co-crystal structure of **42** from the previous study, the authors were able to conceptualize and subsequently synthesize a series of hybrid molecules represented here by compound **45** (Figure 12 and Figure 13H). After demonstrating dose-dependent effector competition across several BRET-based cellular RAS/effector PPI assays and inhibition of pAKT and pERK, **45** was also shown to inhibit the cell viability of DLD-1 cells with an IC_50_ of 5 µM.

Finally, a third class of RAS:effector PPI inhibitors have been reported by Revolution Medicines. Their clinical-stage RAS^MULTI^ and KRAS^G12C^-targeted compounds RMC-6236 and RMC-6291 are purported to work as molecular glues, recruiting the chaperone cyclophilin A to bind KRAS [121]. The binding location of cyclophilin A is such that it blocks RAF binding to KRAS and the resulting downstream signaling [122]. To date, numerous presentations and posters describing this binding have been published [123], while a detailed mechanistic and structural characterization for specific molecules has not yet been described.

## 7. Conclusions

A great deal of signaling within the MAPK/RAS pathway is reliant on protein–protein interactions, and many of the members of the pathway are not traditional enzymes, thus drugging the orthosteric active site is not possible. Additionally, there are some proteins in this pathway where drugging the active site is not desirable. For these reasons, disruption and/or modulation of the PPIs within the MAPK/RAS pathway becomes an attractive axis of drugging the pathway. The discovery and development of small molecules that can target and disrupt these interactions has posed a significant challenge to the scientific community due to the high surface area within the interaction surface between two proteins, the (usually) concomitant tight binding of the protein partners, and the necessity for a well-defined groove or pocket in close proximity to the PPI of the protein partners. Herein, several approaches have been discussed to disrupt PPIs within the RAS/MAPK pathway, harnessing innovative platforms or strategies to find the first toehold of chemical matter. However, there are still plenty of opportunities for growth and improvement to these methodologies. Within this ever-growing field, we look forward to the new technologies and approaches that researchers will utilize to help combat KRAS-mutant driven cancers in the future.

## Data Availability

No new data were created or analyzed in this study. Data sharing is not applicable to this article.
